# Research Progress on Targeted Therapies for Pemphigus Vulgaris and Bullous Pemphigoid: Mechanisms, Opportunities and Challenges

**DOI:** 10.3390/ijms27198779

**Published:** 2026-09-30

**Authors:** Wang-Fei Mo, Ya-Gang Zuo

**Affiliations:** 1 Department of Dermatology, State Key Laboratory of Complex Severe and Rare Diseases, Peking Union Medical College Hospital, Chinese Academy of Medical Sciences & Peking Union Medical College, Beijing 100730, China; 202101030@student.pumc.edu.cn; 2 Chinese Academy of Medical Sciences & Peking Union Medical College, Beijing 100005, China

**Keywords:** pemphigus vulgaris, bullous pemphigoid, targeted therapy, biological agent, small-molecule drug, CAAR-T therapy

## Abstract

Autoimmune bullous diseases (AIBDs) are a group of severe disorders characterized by autoantibodies that attack structural proteins within the skin/mucosa, leading to blister and bullae formation. Pemphigus vulgaris (PV) and bullous pemphigoid (BP) are among the most prevalent representative AIBDs. Traditional treatments involving corticosteroids and immunosuppressive agents are often associated with substantial adverse effects and a high rate of relapse. In recent years, the therapeutic landscape has evolved toward precision targeted therapies, encompassing biological agents, small-molecule drugs, and chimeric autoantibody receptor T cells. These novel interventions act on specific pathogenic targets, offering the potential to improve both efficacy and safety. This narrative review aims to summarize the latest progress in targeted therapies for PV and BP, compare the mechanisms of action, efficacy, and safety profiles of various treatments, and highlight current challenges and future research directions. In addition, therapeutic considerations for patients with concomitant psoriasis are discussed, given the increasing recognition of this comorbidity in clinical practice and the growing number of case reports describing the use of targeted agents in this specific population. Ultimately, the insights presented herein are intended to optimize clinical management strategies and guide future drug development.

## 1. Introduction

Autoimmune bullous diseases (AIBDs) refer to a group of diseases characterized by blisters and erosions on the skin and/or mucous membranes, including pemphigus and pemphigoid diseases [1]. Their pathogenesis involves autoantibodies that target structural proteins of the skin, thereby impairing cell–cell adhesion and leading to blister formation and tissue damage [1,2,3,4]. Pemphigus is mainly mediated by antibodies against desmoglein (Dsg), which disrupt desmosomal adhesion and cause intraepidermal blistering [4,5]. Pemphigoid diseases, on the other hand, are primarily driven by antibodies directed against proteins of the basement membrane zone (BMZ), such as bullous pemphigoid antigen 1 (BPAg1, also known as BP230) and bullous pemphigoid antigen 2 (BPAg2, also known as BP180), impairing dermal-epidermal adhesion and leading to subepidermal blister formation [2,6].

Pemphigus consists of several subtypes, including pemphigus vulgaris (PV), pemphigus foliaceus (PF), pemphigus herpetiformis, pemphigus erythematosus, paraneoplastic pemphigus, drug-induced pemphigus, and immunoglobulin A (IgA) pemphigus [7]. Among these, PV and PF are the most common subtypes [8]. Accordingly, this review focuses specifically on PV. PV is characterized by the presence of both mucosal and cutaneous lesions and is generally more severe than PF [9]. Epidemiological data indicate that the incidence of PV varies from 0.098 to 5 cases per 100,000 person-years, with the mean age of onset between 36.5 and 71 years. The female-to-male prevalence ratio ranges from 0.46 to 4.4 [10]. Several factors have been implicated in the pathogenesis of PV, including certain immunomodulatory drugs [11], malignancies, autoimmune/inflammatory dermatological conditions [12], and genetic susceptibility involving human leukocyte antigen alleles [13,14].

Pemphigoid diseases encompass bullous pemphigoid (BP), linear IgA bullous dermatosis, pemphigoid gestationis, mucous membrane pemphigoid, lichen planus pemphigoides, and dermatitis herpetiformis [15]. This review focuses specifically on BP. Among all pemphigoid diseases, BP exhibits the highest incidence [16], with global rates varying across countries [17], ranging from 1.8 to 7.63 cases per 100,000 person-years [6,16,18], and showing an increasing trend over time [17]. BP predominantly affects individuals aged over 70 years, and the rising incidence may be attributable to age-related immune dysregulation. Other recognized risk factors implicated in BP pathogenesis include genetic susceptibility [19], comorbidities such as neurological disorders (e.g., Parkinson’s disease and dementia) and metabolic diseases (e.g., diabetes), the use of certain medications such as gliptins, immune checkpoint inhibitors (e.g., programmed cell death protein 1 [PD-1] and programmed cell death ligand 1 [PD-L1] inhibitors) [20,21,22], biological agents, loop diuretics, and penicillins [23], as well as physical triggers like radiotherapy, burns, ultraviolet exposure, and surgical procedures [6,15,24]. Both PV and BP clinically manifest with pruritus and pain, significantly impairing patients’ quality of life and may even be life-threatening [3]. Diagnosis typically relies on a combination of clinical features, histopathological examination, direct/indirect immunofluorescence, and serological detection of autoantibodies [1,6].

Traditional therapies, mainly corticosteroids and immunosuppressive agents, are frequently associated with a considerable burden of adverse effects [6,9]. These include metabolic disturbances, hepatic or renal impairment, hematopoietic suppression, an increased risk of infection, and high relapse rates [25,26]. Such limitations underscore the urgent need for refined approaches that can target specific molecules or cells to minimize the harms of broad-spectrum immunosuppression. In recent years, targeted therapies, including biological agents, small-molecule drugs, and chimeric autoantibody receptor T (CAAR-T) cells, have gained significant traction. By precisely interfering with key nodes in the disease-related pathways, these therapies hold promise for improving efficacy and safety. For instance, biological agents such as rituximab (RTX) and dupilumab are already in clinical use; small-molecule drugs, like Janus kinase (JAK) inhibitor and Bruton’s tyrosine kinase (BTK) inhibitor, have demonstrated powerful therapeutic potential; and CAAR-T therapy is currently under clinical investigation for PV. Overall, the therapeutic landscape for AIBDs is shifting from broad immunosuppression toward precision-targeted interventions, driven by the ongoing discovery of novel targets and drug candidates, along with deeper exploration into personalized and combination treatment strategies. However, the available evidence remains fragmented, with a lack of systematic integration and comparative analysis, which inevitably complicates clinical decision-making.

As the coexistence of psoriasis with AIBDs is increasingly recognized in clinical practice, the management of patients with this comorbidity presents distinct therapeutic challenges [27,28]. Several targeted agents effective in psoriasis, including tumor necrosis factor (TNF)-α inhibitors, secukinumab, ixekizumab, ustekinumab, and guselkumab, have been investigated for use in patients with AIBDs. These agents probably act on shared inflammatory pathways implicated in both conditions. However, their paradoxical effects emphasize the need for careful benefit–risk assessment when applying to this specific patient subset.

In this review, we summarize recent advances in targeted therapies for PV and BP, analyze their specific mechanisms of action, clinical efficacy, and safety profiles, with particular attention to drugs applicable in the comorbidity of PV/BP and psoriasis. Furthermore, we outline existing challenges and emerging research directions. We aim to offer a practical framework that can facilitate the optimization of clinical management strategies and provide insights for future drug development. However, it is acknowledged that for many novel therapeutics, the available evidence remains limited to uncontrolled studies or case reports, underscoring the pressing need for more rigorous clinical investigation.

## 2. Methods

To identify the relevant literature and clinical trials regarding targeted therapies for PV and BP, a comprehensive search was conducted in the PubMed and ClinicalTrials.gov databases for records published between 1 January 2000, and 14 September 2026. The search was restricted to English-language publications.

The search strategy utilized a combination of disease-related terms (“pemphigus vulgaris” OR “bullous pemphigoid” OR “pemphigus” OR “pemphigoid, bullous” OR “autoimmune bullous disease*” OR “AIBD*”) and therapy-related terms (“targeted therap*” OR “biological agent*” OR “biological product*” OR “biologics” OR “small-molecule drug*” OR “chimeric autoantibody receptor T cell*” OR “CAAR-T” OR “rituximab” OR “ofatumumab” OR “veltuzumab” OR “ocrelizumab” OR “belimumab” OR “telitacicept” OR “efgartigimod” OR “dupilumab” OR “omalizumab” OR “stapokibart” OR “bertilimumab” OR “nomacopan” OR “Janus kinase inhibitor*” OR “JAK Inhibitor*” OR “Bruton’s tyrosine kinase inhibitor*” OR “BTK Inhibitor*” OR “phosphodiesterase-4 inhibitor*” OR “PDE4 Inhibitor*” OR “spleen tyrosine kinase inhibitor*” OR “Syk Inhibitor*” OR “tumor necrosis factor-alpha inhibitor*” OR “TNF-α Inhibitor*” OR “secukinumab” OR “ixekizumab” OR “ustekinumab” OR “guselkumab”).

For the main text of the narrative review, references were selected according to their relevance to the therapeutic mechanism, clinical study design, recency, and contribution to the interpretation of efficacy and safety. To construct the summary tables, therapies and diseases were queried on ClinicalTrials.gov preferably. A hierarchical selection approach was applied: for therapies with available clinical trials or large cohort studies, corresponding individual case reports were generally excluded to avoid excessive detail. Conversely, for novel therapies lacking high-level evidence, representative case series, case reports, or preclinical studies were included in the tables. During the screening process for the tables, a total of 47 candidate clinical trials, cohort studies, and case series were initially identified for potential inclusion. After excluding two studies with unknown status, two duplicate records, one expanded access program, and one irrelevant study, 41 trials/cohort studies/case series were ultimately described in the summary tables.

## 3. Pathogenic Mechanisms

Although PV and BP have distinct clinical and pathological features, they both belong to the spectrum of AIBDs and share certain core pathogenic mechanisms. Both diseases are primarily driven by autoantibodies, despite targeting different antigens and involving disease-specific immune pathways. In each condition, T cells play a critical regulatory role during immune dysregulation: they help orchestrate B cell activity, promote autoantibody production, and modulate cytokine networks, collectively fueling disease progression [29,30]. The pathways and key cytokines involved in these mechanisms provide a rational foundation for the development of targeted therapies.

### 3.1. Pathogenic Mechanisms of PV

**Figure 1:**
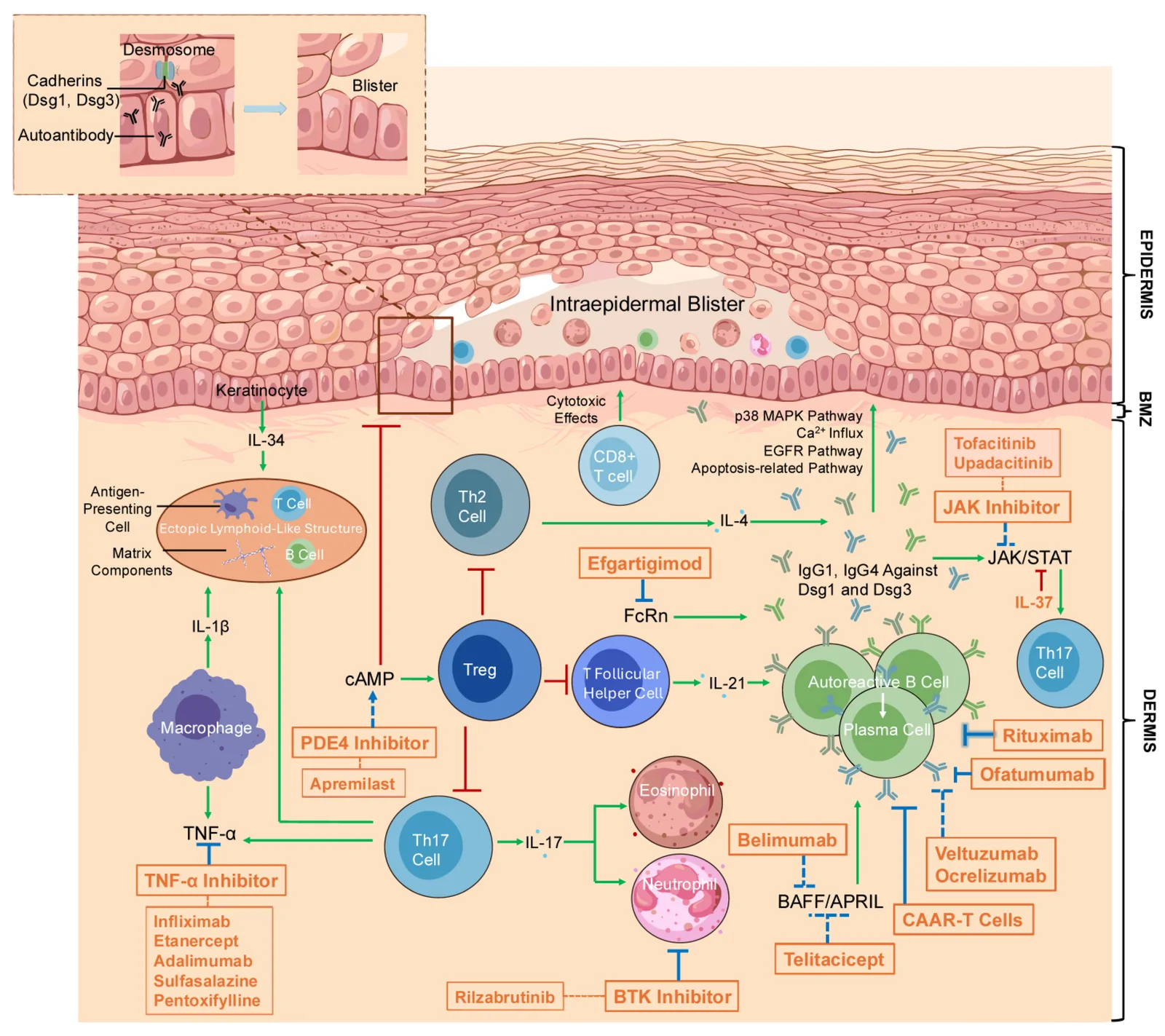
An illustrated overview of the pathogenic mechanisms underlying pemphigus vulgaris, integrated with established and future treatment modalities. Meaning of the symbols shown in the diagram: activation (arrow), inhibition (T-bar), therapies (blue), established therapies (solid line with fluorescence), experimental therapies (solid line), and therapies with weaker evidence (dashed line). Dsg—desmoglein; BMZ—basement membrane zone; IL—interleukin; TNF-α—tumor necrosis factor-α; CD—cluster of differentiation; Th—T helper; Treg—regulatory T cell; cAMP—cyclic adenosine monophosphate; PDE4—phosphodiesterase-4; FcRn—neonatal Fc receptor; p38 MAPK—the p38 mitogen-activated protein kinases; EGFR—epidermal growth factor receptor; IgG—immunoglobulin G; JAK—Janus kinase; STAT—signal transducer and activator of transcription; BTK—Bruton’s tyrosine kinase; BAFF—B cell activating factor; APRIL—a proliferation inducing ligand; CAAR-T cells—chimeric autoantibody receptor T cells.

The core pathogenesis of PV involves autoantibodies directed against Dsg, acting in concert with immune dysregulation mediated by T cells to form a multifactorial disease process (Figure 1).

#### 3.1.1. B Cells and Autoantibodies

Autoreactive B cells that escape immune tolerance produce pathogenic autoantibodies, predominantly IgG1 and IgG4, mainly targeting Dsg3 and Dsg1. These antibodies disrupt desmosomal adhesion, leading to blister formation [31,32,33]. Moreover, memory B cells and long-lived plasma cells often contribute to relapse, which further underscores B cells as a critical therapeutic target. This rationale underpins the use of B cell depleting therapies like RTX, which have proven effective in alleviating the disease.

#### 3.1.2. Antibody-Mediated Signaling Pathway Activation

In addition to directly interfering with the function of Dsg, binding of IgG autoantibodies to Dsg can aberrantly activate downstream signaling pathways such as JAK/signal transducer and activator of transcription (STAT) pathway, the p38 mitogen-activated protein kinases (p38 MAPK) pathway [32,34,35], calcium ion influx [33], the epidermal growth factor receptor pathway, and apoptosis-related pathways, further impairing intercellular connections [36]. These related pathways harbor potential therapeutic targets. For instance, IgG can upregulate histone deacetylase 3 via the p38 MAPK activation, which inhibits Kruppel-like-factor 5 transcription and subsequently downregulates Dsg3 expression [34]. Therefore, inhibitors targeting histone deacetylase 3 may hold therapeutic potential.

#### 3.1.3. T Cells and Cytokines

Upon recognizing Dsg3, cluster of differentiation 4+ (CD4+) T cells facilitate B cell differentiation and the production of pathogenic autoantibodies via cytokines such as interleukin-4 (IL-4) and IL-21. Within this network, T helper (Th) 2 cells secrete IL-4 to promote antibody class switching to IgG4; Th17 cells release IL-17 to exacerbate local inflammation; T follicular helper cells support B cell responses via IL-21; while impaired regulatory T cell (Treg) function undermines immune tolerance [29,37]. CD8+ T cells may also participate in tissue damage through cytotoxic effects [38].

Additionally, IL-37 can inhibit the STAT3 signaling pathway, suppress Dsg3 internalization, and reduce keratinocyte dissociation, thereby attenuating pathological injury [39]. TNF-α triggers multiple intracellular signaling pathways that regulate inflammation, apoptosis, and immune homeostasis [40,41]. Therefore, targeting specific T cell subsets or key cytokines (such as IL-17, TNF-α) and enhancing the IL-37 signaling pathway may provide immune intervention strategies for PV.

#### 3.1.4. Ectopic Lymphoid-like Structure

Ectopic lymphoid-like structures or skin tertiary lymphoid structures are commonly found in chronic skin lesions of PV, and their histological characteristics are similar to those of lymph nodes, including T cells, B cells, antigen-presenting cells, and matrix components [42,43]. Single-cell research confirmed that Dsg-specific B cells, clonally expanded antibody-secreting cells, and T cells with dual T cell receptors were enriched in the skin tertiary lymphoid structures, supporting local autoantibody production and class switching [44,45]. Mechanistically, IL-1β-secreting macrophages are the dominant myeloid subpopulation, which cooperate with IL-34 secreted by keratinocytes to drive ectopic lymphoid-like structures formation through inflammatory and chemotactic pathways [46]. At the same time, Th17 cells are involved in B cell recruitment and germinal center-like responses [44]. These structures not only reflect the degree of local immune activation, but also directly participate in the chronicity of the disease and the continuous production of autoantibodies.

### 3.2. Pathogenic Mechanisms of BP

**Figure 2:**
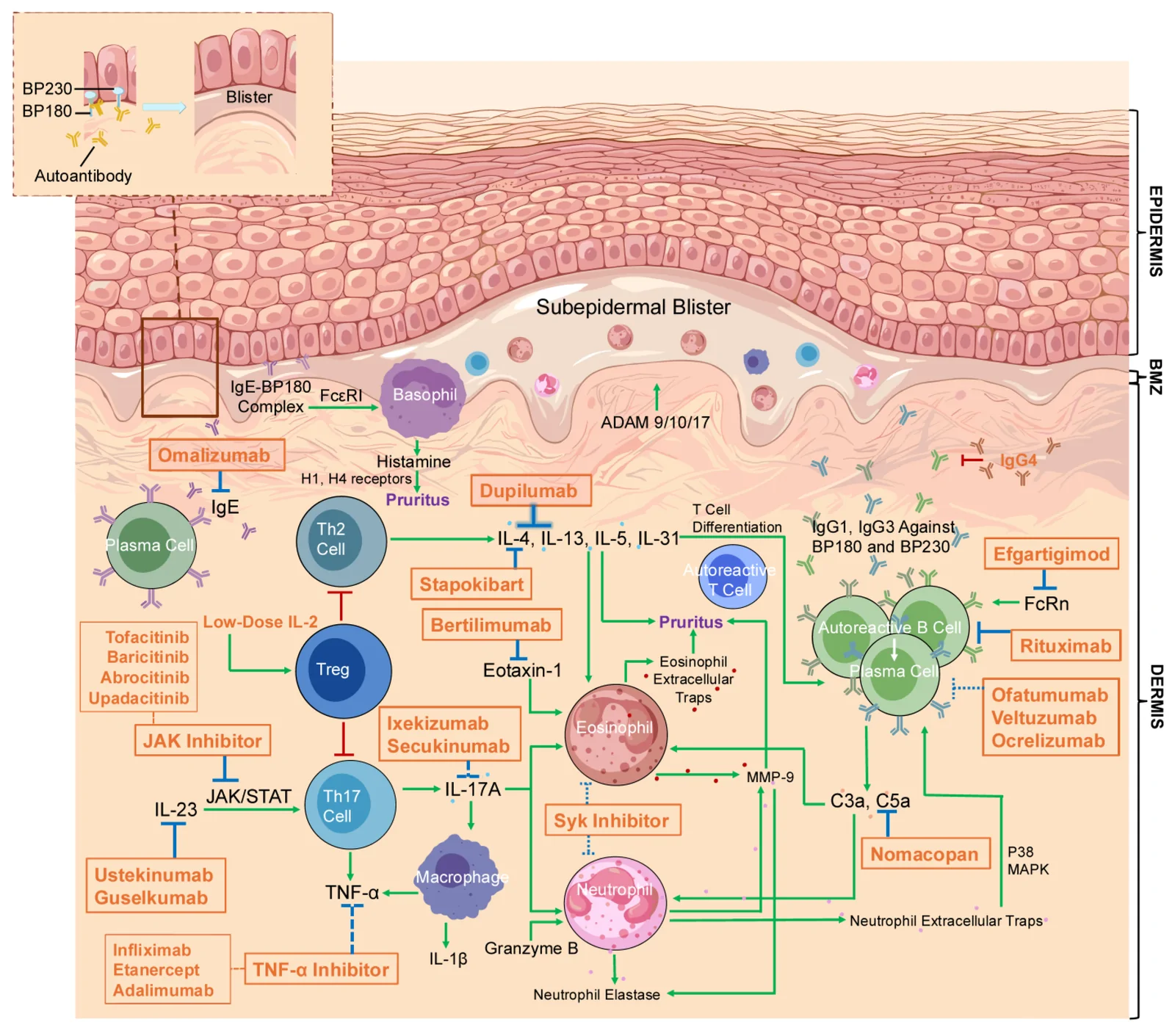
An illustrated overview of the pathogenic mechanisms underlying bullous pemphigoid, integrated with established and future treatment modalities. Meaning of the symbols shown in the diagram: activation (arrow), inhibition (T-bar), therapies (blue), established therapies (solid line with fluorescence), experimental therapies (solid line), and therapies with weaker evidence (dashed line). BP230—bullous pemphigoid antigen 1; BP180—bullous pemphigoid antigen 2; IgE—immunoglobulin E; FcεRI—high-affinity receptor for IgE; Syk—spleen tyrosine kinase; ADAM—a disintegrin and metalloproteinase; MMP-9—matrix metalloproteinase-9; C3a—complement component 3a; C5a—complement component 5a.

The pathogenesis of BP is multifactorial and multistep. The most important step is the binding of autoantibodies (predominantly IgG and IgE) to the hemidesmosomal proteins BP180 and BP230 at the BMZ. This interaction not only triggers complement activation and inflammatory cell infiltration but also amplifies inflammation via protease-mediated degradation and cytokine networks [47]. Collectively, these processes culminate in subepidermal blister formation and the development of intense pruritus (Figure 2).

#### 3.2.1. Autoantibodies and Complement Activation

Pathogenic autoantibodies primarily target the NC16A domain of BP180. Among them, IgG1 and IgG3 can activate the classical complement pathway, leading to the generation of chemotactic factors such as complement component 3a and complement component 5a. These factors recruit eosinophils and neutrophils to the BMZ, thereby contributing to the characteristic linear complement component 3 deposition [2,6,48,49,50]. Deficiency in complement component 5 (C5) has been shown to markedly reduce disease activity in a mouse model, highlighting the pivotal role of complement activation [51]. In contrast, IgG4 may exert an inhibitory effect by competing with IgG1 and IgG3 for epitope binding [52]. Beyond its role in blister formation, IgE also contributes to pruritus. More specifically, IgE-BP180 complexes bind to the high-affinity receptor for IgE (FcεRI) on basophils, promoting the release of histamine which evokes pruritus via H1 and H4 receptors activation on the cutaneous sensory neurons [53,54]. These mechanisms indicate that intervention strategies targeting C5 or IgE signaling pathways hold great therapeutic potential.

#### 3.2.2. Inflammatory Cells and Proteases

Eosinophils and neutrophils are the main effector cells involved in inflammation and blister formation in BP. Eosinophils can directly damage basal keratinocytes, affect complement system balance, and participate in pruritus through mechanisms such as degranulation, release of eosinophil extracellular traps and matrix metalloproteinase-9 (MMP-9), and interaction with IL-13 [55,56]. Upon recruitment and activation, neutrophils release neutrophil elastase, MMP-9, and neutrophil extracellular traps. Neutrophil elastase directly degrades extracellular matrix components of the BMZ, whereas MMP-9 enhances neutrophil elastase activity. In addition, neutrophil extracellular traps can promote B cell differentiation into plasma cells via the p38 MAPK pathway [57].

Furthermore, granzyme B amplifies the inflammatory response through multiple mechanisms: direct proteolysis, enhancing IL-1α activity, promoting IL-8 secretion by keratinocytes, recruiting neutrophils, and cleaving C5 [58]. A disintegrin and metalloproteinase (ADAM) family (e.g., ADAM 9/10/17) also plays a key role in BP pathogenesis through directly cleaving the 120 kDa ectodomain of BP180 [59], downregulating BP180 expression in keratinocytes [59], mediating B cell differentiation [60], and enhancing complement activation [55,58,59,60].

Therefore, targeting these effector cells and their secreted key molecules such as neutrophil elastase, granzyme B or ADAM 9/10/17 is expected to become a novel therapeutic strategy for BP.

#### 3.2.3. T Cells and Cytokines

In the peripheral blood and skin lesions of BP patients, the number of Tregs is significantly reduced and their functions are impaired, resulting in a loss of immune tolerance [61,62]. This defect leads to weakened inhibition of Th2, Th17 and follicular helper T cell, driving the production of autoantibodies against BP180 and BP230 [62,63]. Low-dose IL-2 can specifically stimulate the expansion and activation of Tregs, enhance their sensitivity to T cell receptor signals, and thus restore the immunosuppressive function [61,64]. A clinical study has shown that in patients with moderate-to-severe BP, low-dose IL-2 combined with glucocorticoid can control disease faster and has good safety [61]. This finding indicates low-dose IL-2 has great potential as an adjuvant therapeutic strategy for BP. The inflammatory environment in BP is predominantly driven by both Th2 and Th17 responses. Th2 cytokines (e.g., IL-4, IL-5, IL-13, IL-31) are closely related to T cell differentiation, autoantibody production, eosinophils activation, and pruritus [53,65,66]. Th17 cells and their characteristic cytokines like IL-17A can promote infiltration of neutrophils and eosinophils, induce neutrophil extracellular traps formation, stimulate macrophages to release IL-1β, upregulate interferon γ-inducible protein-10 and MMP-9 expression, and are associated with disease relapse and potential glucocorticoid resistance [37,67,68,69,70]. Therefore, simultaneous targeting of the Th2 and Th17 pathways (such as the combination of anti-IL-4/IL-13 and anti-IL-17 agents) could theoretically improve therapeutic efficacy. Nevertheless, this combination strategy may also raise the risk of adverse events such as infections.

#### 3.2.4. Endocytosis and Signaling Pathways

Beyond immune-mediated damage, the binding of autoantibodies to BP180 can induce keratinocyte endocytosis of this antigen, which then activates intracellular signaling to promote the release of pro-inflammatory cytokines like IL-6 and IL-8, thus non-immunologically weakening the BMZ structure [71]. At the signaling pathway level, the TNF superfamily member 12/Fn14 axis promotes disease progression by activating the extracellular signal-regulated kinase/nuclear factor kappa-B pathway to downregulate BP180 expression and induce protease production [59]. The JAK/STAT pathway, serving as a shared downstream pathway for multiple cytokines (IL-4, IL-6, IL-23, etc.), is widely involved in regulating the differentiation of T cells and represents an important therapeutic target [37,72,73,74]. These abnormalities in non-complement-dependent intracellular signaling pathways provide new therapeutic targets for BP treatment (such as JAK inhibitors).

#### 3.2.5. Immune Checkpoints

Upregulated expression of PD-1 and T cell immunoglobulin and mucin-domain-containing molecule 3 on CD8+ T cells and macrophages have been found in BP lesions [75]. This suggests that dysregulation of immune checkpoint pathways is involved in BP pathogenesis. Notably, the clinical use of immune checkpoint inhibitors for cancer treatment has been found to induce BP [22,76,77]. Therefore, exploring how to utilize checkpoint receptor agonists to restore immune balance offers a novel therapeutic strategy for BP.

## 4. Biological Agents

### 4.1. PV (Table 1)

#### 4.1.1. Rituximab (RTX)

RTX is a chimeric murine/human anti-CD20 monoclonal antibody. It can eliminate autoreactive B cells and reduce the production of anti-Dsg1/Dsg3 antibodies, thereby markedly ameliorating skin lesions and promoting blister healing [31,78,79]. It was granted approval by the US Food and Drug Administration (FDA) for the treatment of moderate-to-severe PV in adults [80].

The optimal dosing strategy for RTX in PV has yet to be clearly established, and current regimens are largely adapted from protocols used in lymphoma and rheumatoid arthritis [81]. According to a study, a single course of standard-dose (1000 mg) could achieve a complete remission rate of approximately 32.4% at 12 months and 43.1% at 36 months with extra infusions [82]. While indirect observations suggest that ultralow-dose (100 mg) and low-dose (500 mg) might exhibit clinical responses similar to standard-dose regimens over 52 weeks with potentially fewer adverse events [81,83], these are highly tentative findings. Direct comparative studies are required to confirm whether their efficacy and safety profiles are truly comparable. Taken together, these data support the development of more personalized, risk-adapted dosing strategies for PV and offer a valuable reference for clinical decision-making.

The advantages of RTX include high complete remission rate and glucocorticoid sparing. However, there are also some limitations such as infusion reactions, increased risk of infections, inadequate response, high relapse rate, and slow onset of action [84,85,86]. Currently, further research is required to optimize its dosage, manage relapse, and reduce costs. Beyond PV, RTX has also been explored in the treatment of BP. According to the European Academy of Dermatology and Venereology guidelines, RTX is recommended as a third-line treatment option for BP [87]. Although it demonstrates clinical efficacy, it appears to be less effective than in PV and is associated with higher rates of relapse and adverse events [88,89]. This indicates that the use of RTX for BP requires greater caution, and further optimization of medication regimens or identification of suitable patient populations is needed in the future.

#### 4.1.2. Ofatumumab (OFA)

Besides RTX, several other anti-CD20 monoclonal antibodies have also demonstrated efficacy in treating PV. OFA is a type I fully human anti-CD20 monoclonal antibody approved for the treatment of relapsing multiple sclerosis and chronic lymphocytic leukemia. It is known to induce stronger antibody-dependent cell-mediated cytotoxicity (ADCC), complement-dependent cytotoxicity (CDC) activity, and apoptotic responses than RTX, which may be attributed to its binding to distinct extracellular epitopes [90,91]. Moreover, because OFA contains a higher proportion of human components, it has been hypothesized that OFA might induce lower immunogenicity and could potentially offer an alternative option when adverse effects occur with RTX [92].

There were two cases, each of which reported a refractory PV patient who was resistant to corticosteroids and refractory to RTX but achieved complete remission following subsequent treatment with OFA [93,94]. Another case report described three patients who experienced PV relapse after achieving clinical remission with RTX. The patients were then treated with subcutaneous OFA in combination with prednisone. Over the 24-week observation period, two of them achieved lesion clearance. No serious adverse events were observed during follow-up [95]. There was also a case report showing that three patients achieved complete remission and discontinued glucocorticoids after the use of OFA [91]. Currently, OFA has been investigated in PV patients through two cohort studies. Both studies revealed that the combination of OFA and glucocorticoids was superior to the traditional treatment in controlling disease, promoting skin healing, and reducing glucocorticoid dose. And the safety profile was favorable as well [96,97]. The current evidence suggests that OFA may be effective in PV, but it still needs to be verified by larger samples.

#### 4.1.3. Veltuzumab

Veltuzumab is a type I humanized anti-CD20 monoclonal antibody characterized by a slower dissociation rate, more potent CDC activity, and more efficient B cell depletion than RTX. In contrast to RTX, both OFA and veltuzumab offer the key advantage of subcutaneous administration [92,98]. A case report described a patient with frequently relapsing PV who developed cytopenia and was refractory to azathioprine, mycophenolate mofetil (MMF), and prednisolone, with a suboptimal response to RTX. This patient achieved complete remission following veltuzumab injections. Upon relapse two years later, complete remission was achieved again after retreatment with the same dose. No injection site reactions or other adverse events were observed following each administration, and the treatment was well-tolerated during the subsequent 35-month follow-up period [99]. This finding indicates that veltuzumab may represent a potential alternative for selected patients in whom RTX proves ineffective or is poorly tolerated.

#### 4.1.4. Ocrelizumab

Ocrelizumab is also a type I humanized anti-CD20 monoclonal antibody that exhibits stronger ADCC and weaker CDC compared to RTX. It appears to deplete CD4+ and CD8+ T cells more profoundly as well [92,100]. This agent is indicated for relapsing or primary progressive multiple sclerosis. Two separate case reports described patients with PV who were refractory to treatments such as azathioprine and corticosteroids, and for whom RTX was not a feasible option. Following ocrelizumab treatment, both patients achieved near-complete remission [101,102]. To date, however, clinical experience with ocrelizumab in PV remains limited. And its long-term safety profile has yet to be established, with infusion-related reaction as one of the commonly observed adverse effects. Well-designed clinical trials are therefore required to support its routine use in PV [103]. Collectively, ocrelizumab shows preliminary therapeutic potential for refractory PV, but its clinical application should remain cautious until robust clinical evidence becomes available.

#### 4.1.5. Belimumab

Belimumab is a fully human monoclonal antibody that targets B cell activating factor (BAFF) and is approved for the treatment of systemic lupus erythematosus [104]. BAFF is a critical factor affecting the survival and differentiation of B cells. In the serum of PV patients, its level is usually increased, which may be associated with the risk of recurrence after RTX therapy [104,105,106]. The mechanism of action of belimumab is to neutralize the soluble BAFF, thus blocking its binding to BAFF receptors and inhibiting the differentiation of B cells and the production of autoantibodies [106]. Its application in the treatment of PV is still in the research stage.

A 60-year-old male PV patient failed to respond to corticosteroid monotherapy and received subcutaneous belimumab 600 mg every two months. Within four months, he showed marked clinical improvement, along with reduced autoantibody titers and corticosteroid requirements [104]. Another report detailed a 27-year-old female PV patient who was unresponsive to previous treatment with prednisone, methylprednisolone, and MMF. She subsequently received intravenous belimumab while continuing methylprednisolone and MMF. Notably, clinical improvement was evident within two weeks. After eight treatment cycles, the patient achieved near-complete remission, the activity of the disease decreased from severe to mild, and the anti-Dsg1/Dsg3 autoantibodies became undetectable. She tolerated belimumab well, and no treatment-related adverse events were reported [107].

So far, evidence supporting the use of belimumab in PV is mainly based on case reports and mechanism studies, and the drug has not yet established its role in standard clinical practice. Future high-quality prospective trials are needed to validate its efficacy and explore optimal therapeutic strategies. One such strategy is sequential therapy with RTX followed by belimumab, as the mechanism of belimumab in treating PV complements that of RTX [106,108]. This approach may be particularly suitable for preventing the resurgence of autoreactive B cells following RTX treatment, which can be driven by elevated BAFF levels.

#### 4.1.6. Telitacicept

Telitacicept is a novel recombinant fusion protein that targets both BAFF and a proliferation inducing ligand (APRIL) and has been approved for the treatment of systemic lupus erythematosus [109]. Its mechanism of action is similar to that of belimumab, but it has a broader inhibitory profile: it can interfere with the maturation of B cells and the survival of plasma cells by binding to BAFF and APRIL [109]. Several cases of PV patients successfully treated with telitacicept have now been reported [110,111,112,113]. These cases indicate that telitacicept may provide a promising alternative treatment for moderate-to-severe patients or those who respond poorly to conventional immunosuppression. It appears to be fast-acting, with a low risk of serious infection. Nevertheless, current data on the application of telitacicept in PV is limited, and most of the clinical evidence is indirect, thus further research is needed to confirm these findings.

#### 4.1.7. Efgartigimod

The neonatal Fc receptor (FcRn) plays a key role in the stabilization and recycling of autoantibodies, thereby prolonging the half-life of IgG. Targeted intervention against FcRn has brought novel therapeutic prospects for various autoimmune diseases [114]. Efgartigimod is a humanized IgG1 Fc fragment derivative designed to target FcRn and accelerate the degradation of pathogenic IgG. It has been approved for the treatment of myasthenia gravis [89].

A case reported a patient with refractory PV who achieved significant clinical improvement after treatment with efgartigimod [115]. A multicenter phase 2 trial (NCT03334058) evaluated efgartigimod in 34 patients with pemphigus (26 with PV, 8 with PF). The drug was well tolerated, produced rapid reductions in total IgG and anti-Dsg antibody levels, and led to early control and complete remission [116]. A subsequent secondary analysis of this trial indicated that, in several patients who responded to efgartigimod, the sustained decrease in autoantibodies and Dsg-specific B cells may point to disease-modifying effects extending beyond the transient clearance of IgG. Furthermore, efgartigimod may carry a low risk of infection by avoiding complete B cell depletion [117]. It was also proposed that efgartigimod may, in the treatment of PV, act directly on keratinocytes to stabilize cell–cell adhesion and improve skin lesions [118]. However, in a randomized, placebo-controlled phase 3 trial (NCT04598451) investigating the efficacy and safety of efgartigimod in 222 adult patients with PV/PF, no statistically significant difference was observed between the efgartigimod and placebo group [119,120].

In addition to its role in the treatment of PV, there is also evidence for efgartigimod in BP. Inhibition of FcRn can reduce the overall IgG and pathogenic autoantibody levels, which has shown therapeutic potential in a mouse model of BP [121]. A randomized, placebo-controlled phase 2/3 clinical study (NCT05267600) evaluating the efficacy, safety, and tolerability of efgartigimod PH20 in BP has been completed. Results showed that after 36 weeks, the efgartigimod treatment group achieved a higher complete remission rate and required lower glucocorticoid doses compared to the placebo group. The phase 3 portion of this trial was not conducted. Another phase 3 clinical trial (NCT05681481) was terminated due to sponsor decision [122]. Although FcRn inhibitors hold promise for immunomodulation in BP, their clinical application still warrants further evidentiary support.

**Table 1:** Landscape of biological agents for PV.

Drug	Target	Regulatory/Clinical Status	Study (Phase)	Status	Characteristics	Main Results	Actual (Estimated) Participants	Level of Evidence *
Rituximab (RTX)	CD20	FDA-approved for adult patients with moderate-to-severe PV	NCT05898308 (Phase 4)	Recruiting (estimated completion in 2032)	Randomized, open-label, controlled trial comparing personalized maintenance therapy vs. standard RTX + corticosteroids in pemphigus	-	(133)	-
			NCT02383589 (Phase 3) [123]	Completed in 2019	Randomized, double-blind, active-comparator, multicenter trial comparing RTX vs. MMF in moderate-to-severely PV (with prednisone or equivalent)	RTX yielded a higher 52-week corticosteroids-free CR rate (40.3% vs. 9.5%), fewer flares, and lower cumulative corticosteroids doses than MMF, but had a higher SAE rate.	135	2
			NCT03790293 (Phase 3) [124]	Completed in 2019	Randomized, open-label, long-term follow-up study of pemphigus patients treated with RTX (NCT00784589)	First-line RTX + prednisone achieved higher 7-year corticosteroids-free disease survival (72.1% vs. 35.3%) and a lower relapse rate (42.2% vs. 83.7%) than prednisone alone.	80	3
			NCT00784589 (Phase 3) [125]	Completed in 2016	Randomized, open-label, multicenter trial comparing RTX vs. standard corticosteroids in pemphigus	First-line RTX + short-term prednisone yielded a higher 24-month corticosteroids-free CR (89% vs. 34%), a lower relapse rate, and fewer SAEs than prednisone alone.	90	2
			NCT01299857 (Phase 3)	Completed in 2012	Open-label, single-arm, long-term follow-up study of severe pemphigus patients treated with RTX (NCT00213512)	No results posted.	22	-
			NCT00213512 (Phase2/3) [126]	Completed in 2008	Non-randomized, open-label study of RTX in corticosteroid-dependent, -resistant, or -contraindicated pemphigus	Single-cycle RTX yielded an 86% CR rate at 3 months with substantial corticosteroids reduction; 45% of CR patients relapse (median 18.9 months). SAEs included pyelonephritis and septicemia.	21	3
			NCT04400994 (Phase 2)	Active, not recruiting (estimated completion in 2026)	Randomized, open-label, active-controlled trial comparing RTX + IVIG vs. RTX monotherapy as first-line treatment in moderate-to-severe pemphigus	-	20	-
			NCT00656656 (Phase 2)	Completed in 2011	Open-label, single-arm study evaluating protein A immunoadsorption + RTX + dexamethasone + AZA in pemphigus	All patients achieved short- and long-term remission up to 43 months; 2 patients experienced treatment-related AEs.	23	4
			NCT06581562 (Phase 1)	Recruiting (estimated completion in 2028)	Open-label, single-arm, single-center study evaluating RTX + allogeneic NK cell therapy in B cell associated autoimmune diseases (including PV)	-	(30)	-
			NCT07769333 (Early phase 1)	Not yet recruiting (estimated completion in 2027)	Quasi-experimental, non-randomized trial comparing RTX + corticosteroids vs. AZA + corticosteroids in moderate-to-severe PV	-	(92)	-
			NCT07457944	Completed in 2026	Retrospective, multicenter case–control study evaluating different RTX dosing regimens (500 mg once, 500 mg twice, 1000 mg twice) in PV/PF	No results posted.	275	-
			NCT06949241	Active, not recruiting (estimated completion in 2026)	Retrospective, single-center case–control study evaluating single-cycle IVIG + RTX in PV/PF	-	76	-
			NCT00960713	Completed in 2012	Prospective, observational cohort study evaluating SAEs of off-label RTX use in various autoimmune diseases (including pemphigus)	No results posted.	35	-
Ofatumumab (OFA)	CD20	FDA-approved for multiple sclerosis and chronic lymphocytic leukemia; investigational/off-label for PV	NCT02613910 (Phase 3)	Terminated (sponsor discontinued for non-safety reasons)	Open-label, single-arm, multicenter, long-term extension study of OFA in PV (NCT01920477)	-	1	-
			NCT01920477 (Phase 3)	Terminated (sponsor discontinued for non-safety reasons)	Randomized, double-blind, placebo-controlled trial evaluating subcutaneous OFA in PV	-	35	-
			NCT06654817 [96]	Completed in 2024	Prospective, observational, multicenter cohort study comparing OFA + corticosteroids vs. AZA + corticosteroids in PV	OFA group achieved faster disease control, a higher 36-week CR rate, and lower cumulative steroid exposure; 2 serious pneumonia cases occurred.	38	3
			-	Published in 2025	Non-randomized, registry-based cohort study evaluating OFA in PV [97]	OFA + glucocorticoids yielded higher 52-week CR rates and lower cumulative corticosteroids exposure without increased drug-related SAEs compared to conventional therapy.	48	3
Veltuzumab	CD20	Investigational for PV	Case report evidence only [99]					5
Ocrelizumab	CD20	FDA-approved for multiple sclerosis; investigational/off-label for PV	Case report evidence only [101,102]					5
Belimumab	BAFF	FDA-approved for systemic lupus erythematosus and lupus nephritis; investigational/off-label for PV	Case report evidence only [104,107]					5
Telitacicept	BAFF and APRIL	NMPA-approved for systemic lupus erythematosus, etc.; investigational/off-label for PV	Case report evidence only [110,111,112,113]					5
Efgartigimod	FcRn	FDA-approved for generalized myasthenia gravis and chronic inflammatory demyelinating polyneuropathy; investigational/off-label for PV/BP	NCT04598477 (Phase 3)	Terminated (sponsor discontinued due to lack of efficacy in NCT04598451)	Open-label, multicenter, follow-up trial of NCT04598451	-	183	-
			NCT04598451 (Phase 3)	Completed in 2023	Randomized, double-blind, placebo-controlled, multicenter trial evaluating efgartigimod PH20 in PV/PF	Failed primary endpoint; 35.5% achieved sustained CR on minimal corticosteroids vs. 30.3% in placebo (*p* = 0.5956).	222	2
			NCT03334058 (Phase 2) [116]	Completed in 2020	Open-label, non-controlled, adaptive-design trial evaluating efgartigimod in mild-to-moderate PV/PF	90% achieved disease control (median 17 days); 64% attained CR on low-dose prednisone (median 92 days).	34	4

* The levels of evidence are determined according to the Oxford Centre for Evidence-Based Medicine grading criteria (2011). PV—pemphigus vulgaris; CD—cluster of differentiation; FDA—Food and Drug Administration; MMF—mycophenolate mofetil; CR—complete remission; SAE—severe adverse event; AE—adverse event; IVIG—intravenous immunoglobulin; AZA—azathioprine; NK—natural killer; PF—pemphigus foliaceus; BAFF—B cell activating factor; APRIL—a proliferation inducing ligand; NMPA—National Medical Products Administration; FcRn—neonatal Fc receptor.

### 4.2. BP (Table 2)

#### 4.2.1. Dupilumab

Dupilumab is a recombinant fully human monoclonal antibody targeting the IL-4 receptor alpha subunit (IL-4Rα). It can block both IL-4 and IL-13 signaling and inhibit the secretion of IL-31 by eosinophils. Through this mechanism, it effectively suppresses Th2-mediated inflammatory responses [127,128,129]. The drug is now FDA-approved for the treatment of BP in adults [130].

In a multicenter retrospective cohort study (NCT05649579) evaluating the efficacy and safety of dupilumab in BP, among 146 patients, 127 achieved disease control within 4 weeks, and 107 had no adverse events. This study suggests that dupilumab is a great option for BP, but also points out that infection risk warrants attention, especially in elderly patients with comorbidities [131]. A summary study further supported these positive findings, with all included systematic reviews consistently affirming the efficacy and favorable safety profile of dupilumab in BP [132]. A phase 2/3 randomized controlled trial (NCT04206553) investigating dupilumab for BP was completed [133]. The results demonstrated that, compared to placebo, dupilumab led to better clinical improvement with a five-fold higher rate of sustained remission. It relieved pruritus faster and reduced corticosteroid requirements.

Numerous other studies have also shown that dupilumab provides rapid and sustained symptom control, particularly in patients with refractory pruritus or moderate-to-severe disease [129,133,134,135,136,137,138]. Moreover, dupilumab is generally well-tolerated with a low incidence of side effects in these patients, which suggests that it might be a suitable option for vulnerable populations who are unable to tolerate conventional therapies [135,139]. However, long-term safety data from large-scale studies remain limited, necessitating further evaluation.

#### 4.2.2. Omalizumab (OMZ)

OMZ is a recombinant humanized monoclonal antibody approved for the treatment of chronic spontaneous urticaria and severe asthma. In recent years, it has demonstrated significant efficacy in the treatment of BP [140]. Its mechanism of action centers on binding to and depleting free IgE in the serum, thereby preventing IgE from interaction with FcεRI. This interruption of the autoimmune cascade helps reduce local inflammation and dermal-epidermal separation [141,142]. Long-term use of OMZ may also suppress the production of IgE, lower the count of peripheral blood eosinophils, and downregulate the expression of FcεRI on inflammatory cells [143]. The first successful use of OMZ in a patient with refractory BP can be traced back to 2009 [144]. Since then, retrospective studies and case reports have confirmed that OMZ can achieve a high complete remission rate in BP patients who are unresponsive or resistant to first-line therapies. It has advantages of a rapid onset of action and a favorable safety profile [88,128,140,141]. However, there are some limitations in the existing retrospective research, such as inconsistent dosing regimens and absence of standardized washout periods. Well-designed prospective trials in the future are essential to define optimized dosing strategies and to assess the durability of response, thereby strengthening the role of OMZ in BP treatment guidelines.

#### 4.2.3. Stapokibart

Stapokibart is a humanized monoclonal antibody developed for the treatment of Th2-mediated inflammatory diseases, such as allergic rhinitis and moderate-to-severe atopic dermatitis [145]. Like dupilumab, it also targets IL-4Rα but binds to a distinct epitope [145,146]. Theoretically, stapokibart holds potential for the treatment of BP. In a single-center retrospective cohort study including 12 BP patients, all patients were treated with stapokibart and tripterygium glycosides, and seven of them were treated with oral corticosteroids at the same time. As a result, all patients achieved disease control, with a median time of 14.5 days, and 91.7% were controlled within 4 weeks [147]. In this study, stapokibart-based combination therapy was associated with rapid short-term disease control and clinical improvement. However, this study is limited by its small sample size, lack of a control group, and confounding concurrent medications (e.g., tripterygium glycosides and corticosteroids), precluding the attribution of efficacy solely to stapokibart. Furthermore, the short follow-up period limits the evaluation of long-term remission durability, relapse rates, and rare adverse events. In another single-center cohort study including 15 elderly moderate-to-severe BP patients, all patients achieved disease control within 4 weeks, of which 86.67% were controlled within 2 weeks, with no serious adverse events reported. However, four patients subsequently experienced disease relapse [148]. Similarly, the patients also used corticosteroids or minocycline in this study. In addition, there was a successful case of using stapokibart to treat dipeptidyl peptidase-4 inhibitor-induced BP in an elderly diabetic patient [149]. Recently, a phase 3 randomized controlled trial (NCT07210554) investigating stapokibart for moderate-to-severe BP is underway. Drawing on the positive outcome observed with stapokibart in BP, the exploration of stapokibart’s efficacy and safety is anticipated to emerge as a notable research direction in the future.

#### 4.2.4. Bertilimumab

Bertilimumab is a fully human monoclonal antibody that targets eotaxin-1 and inhibits the migration of eosinophils to the skin. A completed phase 2 trial (NCT02226146) evaluated the efficacy and safety of this agent in nine patients with moderate-to-severe BP. After 13 weeks of treatment, the patients showed an 81% reduction in Bullous Pemphigoid Disease Area Index, along with a decreased need for corticosteroid [150]. These findings suggest that bertilimumab may represent an effective therapeutic option for BP. However, the trial was limited by its small sample size and lack of a control group, and long-term safety data remained to be established.

#### 4.2.5. Nomacopan

Nomacopan is a recombinant protein that can bind and inhibit both C5 and leukotriene B4, thus blocking their pro-inflammatory activities. A phase 2a trial (NCT04035733) involving nine patients with BP reported that nomacopan had encouraging preliminary efficacy, particularly in elderly patients. Most patients showed meaningful clinical improvement, and some achieved near-complete remission. In addition, the treatment was well-tolerated, and no drug-related serious adverse event was observed [151]. A placebo-controlled phase 3 trial (NCT05061771), designed to further assess the efficacy and safety of nomacopan in combination with oral corticosteroids in BP, was ultimately withdrawn due to strategic resource allocation decisions.

**Table 2:** Landscape of biological agents for BP.

Drug	Target	Regulatory/Clinical Status	Study (Phase)	Status	Characteristics	Main Results	Actual (Estimated) Participants	Level of Evidence *
Efgartigimod	FcRn	FDA-approved for generalized myasthenia gravis and chronic inflammatory demyelinating polyneuropathy; investigational/off-label for PV/BP	NCT05681481 (Phase 3)	Terminated (sponsor decision)	Open-label, single-arm long-term extension study of NCT05267600	-	64	-
			NCT05267600 (Phase 2/3)	Completed in 2024	Randomized, double-blind, placebo-controlled, multicenter trial comparing efgartigimod PH20 + prednisone vs. placebo + prednisone in adult patients with BP	16.0% in efgartigimod group achieved corticosteroids-free CR for ≥8 weeks vs. 4.2% in placebo.	98	2
Dupilumab	IL-4Rα	FDA-approved for adult patients with BP	NCT04206553 (Phase 2/3) [133]	Completed in 2025	Randomized, double-blind, placebo-controlled, multicenter trial comparing dupilumab + corticosteroids vs. placebo + corticosteroids in BP	18.2% in dupilumab group achieved sustained remission at 36 weeks vs. 4.0% in placebo.	106	2
			NCT05649579 [131]	Completed in 2023	Retrospective, multicenter cohort study evaluating dupilumab in BP	87.0% achieved disease control within 4 weeks (median 14 days); 64-week CR rate was 62.5%; 73.3% reported no AEs.	146	4
Omalizumab (OMZ)	IgE	FDA-approved for chronic spontaneous urticaria, etc.; investigational/off-label for BP	NCT00472030 (Phase 4)	Completed in 2010 (actually terminated due to poor enrolment)	Open-label, single-arm case series evaluating OMZ in BP	-	2	-
			NCT04128176 (Phase 3)	Withdraw (PI did not pursue the study; the study was not IRB-approved)	Open-label, non-controlled, single-arm, single-center study evaluating RTX + OMZ in BP	-	0	-
			-	Published in 2025	Retrospective, multicenter cohort study comparing OMZ vs. dupilumab in BP in a real-world setting [152]	Disease control and CR on minimal therapy were achieved by 90.6% and 71.0% in dupilumab group, and 77.8% and 64.7% in OMZ group, respectively.	58	3
Stapokibart	IL-4Rα	NMPA-approved for seasonal allergic rhinitis, etc.; investigational/off-label for BP	NCT07210554 (Phase 3)	Not yet recruiting (estimated completion in 2028)	Randomized, double-blind, placebo-controlled trial evaluating stapokibart in moderate-to-severe BP	-	(200)	-
			-	Published in 2026	Real-world retrospective, single-center cohort study evaluating stapokibart-based combination therapy in BP [147]	All patients achieved disease control (median 14.5 days) and BPDAI = 0 (median 65.5 days); 58.3% reached CR on minimal therapy (median 176 days).	12	4
			-	Published in 2026	Retrospective, single-center, uncontrolled case series evaluating stapokibart in elderly with moderate-to-severe BP [148]	All patients achieved disease control within 4 weeks; BPDAI decreased from 78.80 to 5.35 at 4 weeks; 26.67% relapsed.	15	4
Bertilimumab	Eotaxin-1	Investigational for BP (orphan drug designation)	NCT02226146 (Phase 2)	Completed in 2018	Open-label, single-arm, proof-of-concept study evaluating bertilimumab in newly diagnosed, moderate-to-extensive BP	85.7% achieved ≥50% improvement (57.1% achieved ≥90%); 66.7% experienced treatment emergent AEs; no anti-drug antibodies detected.	9	4
Nomacopan	C5 and Leukotriene B4	Investigational for BP (orphan drug designation)	NCT05061771 (Phase 3)	Withdraw (sponsor discontinued due to strategic resource allocation decisions)	Randomized, partial/double-blind, placebo-controlled study evaluating nomacopan + corticosteroid in adult patients with BP	-	0	-
			NCT04035733 (Phase 2) [151]	Completed in 2020	Open-label, single-arm study evaluating nomacopan in adult patients with mild-to-moderate BP	Mean BPDAI decreased from 32.0 to 19.6 at 42 days; no treatment-related SAEs occurred.	9	4

* The levels of evidence are determined according to the Oxford Centre for Evidence-Based Medicine grading criteria (2011). BP—bullous pemphigoid; IL-4Rα—interleukin-4 receptor alpha subunit; IgE—immunoglobulin E; PI—principal investigator; IRB—institutional review board; RTX—rituximab; BPDAI—bullous pemphigoid disease area index; C5—complement component 5.

## 5. Small-Molecule Drugs for PV and BP (Table 3)

### 5.1. JAK Inhibitor

The JAK/STAT signaling pathway plays a critical role in the pathogenesis of both PV and BP [153]. Accumulating case reports have revealed that JAK inhibitors can achieve rapid disease control and reduce corticosteroid requirements. The JAK family comprises four members: JAK1, JAK2, TYK2, and JAK3. Notably, different JAK inhibitors display varying selectivity for these subtypes. Among these, tofacitinib (targeting JAK1 and JAK3) and upadacitinib (JAK1-selective) have shown efficacy in refractory PV. In the context of BP, favorable outcomes have been reported with tofacitinib, baricitinib (targeting JAK1 and JAK2), abrocitinib (JAK1-selective), and upadacitinib [37,154].

Although JAK inhibitors have demonstrated remarkable efficacy in clinical reports and hold benefits such as oral administration and suitability for patients with concomitant psoriasis, they are also associated with potential adverse effects, including malignancies, thrombosis, cardiovascular events, and infections [89,155]. For vulnerable groups, like the elderly and those with a history of smoking, it is especially recommended to use the drug with caution [37,154].

Looking ahead, ongoing research into JAK inhibitors is likely to focus on several key areas: elucidating the specific roles of different JAK subtypes in PV and BP, identifying predictive biomarkers of therapeutic response, and developing combination treatment strategies to enhance efficacy while minimizing toxicity. However, interventions of JAK inhibitors still need to balance their anti-inflammatory effects with the risk of systemic immunosuppression.

### 5.2. BTK Inhibitor

BTK is an enzyme involved in both innate and adaptive immune responses. Since it participates in the proliferation and differentiation of B cells and promotes the production of inflammatory cytokines in myeloid cells, it has become an attractive therapeutic target for immune-mediated diseases and B cell malignancies [156]. BTK inhibitors can not only target the pathological processes of PV and BP, but also have the advantages of multi-pathway intervention, rapid onset of action and a favorable safety profile [156].

Rilzabrutinib is a highly selective, reversible covalent BTK inhibitor. It exhibits lower off-target effect and systemic exposure [103]. A single-arm, multicenter phase 2 clinical trial (NCT02704429) reported that rilzabrutinib, either as monotherapy or in combination with low-dose corticosteroids, achieved rapid disease control in PV patients, with efficacy improving over the course of treatment. Adverse events were predominantly mild to moderate and reversible [157]. Commonly observed adverse reactions included bleeding, rash, diarrhea, atrial fibrillation, infections, and hypertension [158]. However, in a subsequent phase 3 randomized controlled trial (NCT03762265), it was found that rilzabrutinib did not demonstrate a statistically significant difference in remission rates compared with placebo, and the study was ultimately terminated [159].

Challenges associated with BTK inhibitors in the treatment of PV include inconsistent individual therapeutic responses, drug resistance, and the management of adverse effects. Furthermore, research progress on BTK inhibitors in BP remains relatively limited compared to PV. Theoretically, BTK inhibitors could also be applicable to BP, but there are currently no reported specific clinical trial data for BP. Building on the experience in PV, exploring the efficacy of BTK inhibitors in BP may represent a potential future research direction.

### 5.3. Phosphodiesterase-4 (PDE4) Inhibitor

PDE4 inhibitors, by elevating intracellular levels of cyclic adenosine monophosphate (cAMP), help maintain the stability of epithelial cell adhesion [160], downregulate pro-inflammatory cytokines, promote Treg responses [161], and reduce autoantibody levels [162]. Apremilast, an oral PDE4 inhibitor, has been utilized in the treatment of dermatological conditions such as psoriasis and has also demonstrated certain efficacy in the management of PV [163,164].

The first reported case utilizing apremilast for refractory PV demonstrated that the drug could significantly improve symptoms, with blisters resolving and skin lesions healing. The therapeutic effect was durable, and no serious adverse events occurred during treatment [161]. Subsequently, another report detailed a patient with PV. The patient was initially misdiagnosed with atypical Behçet’s disease and unresponsive to colchicine. Following a switch to apremilast, the patient experienced marked symptomatic improvement. Notably, the patient relapsed rapidly within days of discontinuation but promptly improved again upon reintroduction [162]. In these cases, apremilast exhibited a favorable safety profile; any adverse effects, such as gastrointestinal discomfort and headache, were generally transient and manageable [161,162]. However, a case report described two patients with moderate-to-severe PV who showed no therapeutic response after 2 months of apremilast therapy, with one even experiencing disease exacerbation [165]. Whether apremilast has reliable efficacy in PV remains uncertain, and more robust evidence is needed.

In theory, PDE4 inhibitors may also alleviate inflammatory damage in BP by modulating cAMP levels. Given the potential efficacy observed in PV, a deeper understanding of how the PDE4/cAMP pathway is differentially regulated in these two diseases may help clarify the potential role of PDE4 inhibitors in BP.

### 5.4. Spleen Tyrosine Kinase (Syk) Inhibitor

An experiment using a fully human model of BP demonstrated that Syk inhibitors could suppress the response of granulocytes to deposited immune complexes, thereby significantly alleviating dermal-epidermal separation [166,167]. This agent does not affect granulocyte migration, overall reactivity, and survival, indicating that its function is selective [166]. To date, there have been no reports on the use of Syk inhibitors in PV models. Nevertheless, the existing preclinical evidence supports the potential clinical translation of Syk inhibitors for BP and highlights their promise as an emerging therapeutic option.

**Table 3:** Landscape of small-molecule drugs for PV and BP.

Drug	Regulatory/Clinical Status	Disease	Study (Phase)	Status	Characteristics	Main Results	Actual (Estimated) Participants	Level of Evidence *
JAK inhibitor	FDA-approved for rheumatoid arthritis, etc.; investigational/off-label for PV/BP	BP	NCT06561256	Completed in 2024	Retrospective, single-center, comparative cohort study evaluating JAK inhibitor + corticosteroids in BP	No results posted.	120	-
			-	Published in 2025	Case series reporting the efficacy of oral tofacitinib in steroid-resistant refractory BP [153]	Mean BPDAI decreased from 82.8 to 22.0 at 1 month, alongside decreased granzyme B levels.	8	4
			-	Published in 2023	Case series reporting the efficacy and safety of tofacitinib in refractory BP [168]	All patients became itch-free within 1 week and maintained CR for 9–14 months; anti-BP180-NC16A IgG and eosinophils levels significantly reduced.	7	4
		PV	Case report evidence only [169,170,171]					5
BTK inhibitor	FDA-approved for immune thrombocytopenia; investigational/off-label for PV	PV	NCT03762265 (Phase 3) [159]	Terminated (sponsor decision based on lack of efficacy)	Randomized, double-blind, placebo-controlled, multicenter study evaluating rilzabrutinib in moderate-to-severe pemphigus	-	131	-
			NCT02704429 (Phase 2)	Completed in 2020	Open-label, single-arm study evaluating rilzabrutinib in newly diagnosed or relapsing PV	51.9% of 27 patients achieved disease control within 4 weeks on low-dose glucocorticoids; 74.1% reported treatment-emergent AEs (11.1% serious).	42	4
PDE4 inhibitor	FDA-approved for psoriasis, etc.; investigational/off-label for PV	PV	Case report evidence only [161,162,165]					5
Syk inhibitor	Investigational for BP (preclinical stage)	BP	Preclinical evidence only [166]					5

* The levels of evidence are determined according to the Oxford Centre for Evidence-Based Medicine grading criteria (2011). JAK—Janus kinase; BP180—bullous pemphigoid antigen 2; IgG—immunoglobulin G; BTK—Bruton’s tyrosine kinase; PDE4—phosphodiesterase-4; Syk—spleen tyrosine kinase.

## 6. CAAR-T Therapy

Based on the breakthrough success of chimeric antigen receptor T cell (CAR-T) immunotherapy in hematologic malignancies, this technology is now being increasingly explored for the treatment of autoimmune diseases. CAAR-T therapy is a technical extension and conceptual innovation of CAR-T therapy. By fusing Dsg3 with the CD137-CD3ζ signaling domain, Dsg3 CAAR-T cells have been engineered for the treatment of PV. Unlike conventional CAR-T cells, which target surface antigens on all B cells and cause broad B cell depletion, Dsg3 CAAR-T cells are designed to specifically recognize B cells expressing the anti-Dsg3 B cell receptor, thereby selectively eliminating autoreactive B cells while theoretically preserving the majority of normal humoral immunity [172]. In a mouse model, experimental pemphigus was induced by transferring a Dsg3 hybridoma cell line. After administration of Dsg3 CAAR-T cells, the mice exhibited reduced serum anti-Dsg3 antibody titers, elimination of autoantibody deposition in the skin and mucous membranes, and prevention of oral mucosal blister formation [173]. A phase 1/2 clinical trial (NCT04422912) evaluating the maximum tolerated dose and safety of Dsg3 CAAR-T in patients with mucosal-dominant PV is currently underway.

Although CAAR-T technology offers a highly targeted and personalized strategy, and holds the speculative potential for long-term remission through the formation of immunological memory, several important uncertainties remain. First, adverse effects observed with conventional CAR-T therapy (including cytokine-release syndrome, neurotoxicity, antigen escape, and on-target/off-tumour toxicity) may also occur with CAAR-T, though their incidence and severity remain to be defined. Second, the durability of response, manufacturing complexity, high costs, and the feasibility of large-scale clinical application are yet to be established. The potential for a curative effect should therefore be regarded as speculative at this early stage, rather than as an established expectation. Compared with other emerging therapies, the clinical evidence for CAAR-T remains limited to preclinical models and an early-phase trial, and further validation through larger, longer-term studies is essential. If ongoing clinical trials demonstrate favorable risk–benefit profiles, CAAR-T cells could ultimately represent a paradigm-shifting therapeutic option for PV. Furthermore, this platform may provide a technological foundation for developing similar antigen-specific cell-based therapies for other pemphigus subtypes or pemphigoid diseases.

## 7. Therapeutic Agents for AIBDs with Concomitant Psoriasis: Opportunities and Pitfalls (Table 4)

**Figure 3:**
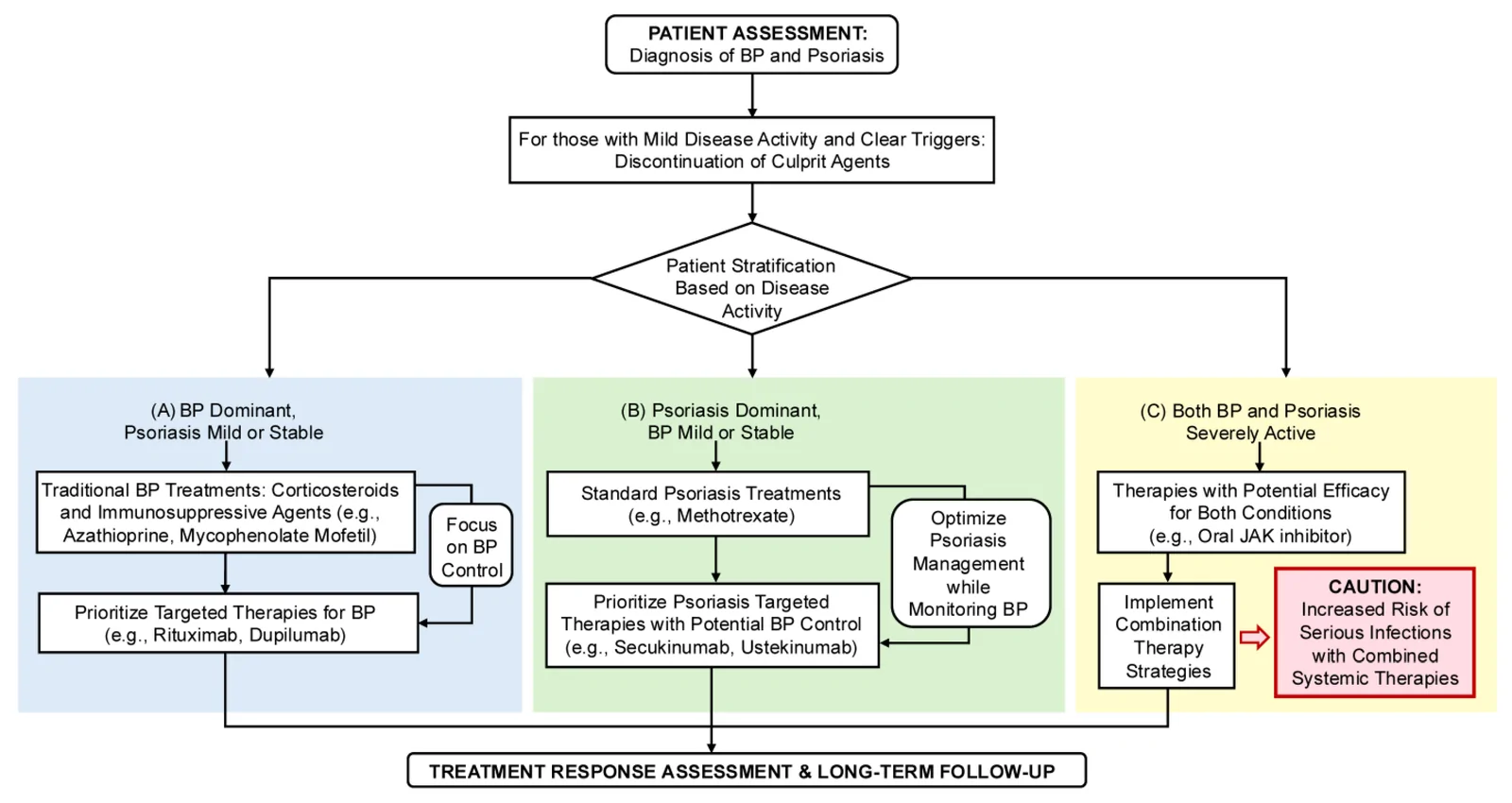
A hypothetical conceptual framework exploring potential therapeutic considerations for coexisting BP and psoriasis. This algorithm has not been prospectively validated and should not be interpreted as a clinical guideline. The proposed approach is based largely on case reports, mechanistic reasoning, and psoriasis/BP treatment extrapolation. Validating this proposed algorithm requires robust clinical trials. Within this conceptual model, patients with both BP and psoriasis are divided into 3 categories: (**A**) BP dominant, psoriasis mild or stable: targeted therapies primarily directed against BP (e.g., rituximab, dupilumab) might be considered. (**B**) Psoriasis dominant, BP mild: psoriasis-targeted agents with reported potential to incidentally control BP (e.g., secukinumab and ustekinumab) could be explored. (**C**) Both are severely active: JAK inhibitors (oral, potentially effective for both) or combination treatment strategies are theoretical options, though they require strict monitoring for infection risks. Mechanistically, the biologics proposed in this flowchart represent possible therapeutic options. However, because paradoxical effects have been reported to occasionally trigger or exacerbate the disease, clinical management necessitates an individualized, multidisciplinary assessment and a highly careful benefit–risk evaluation for each patient. BP—bullous pemphigoid.

Studies have shown that psoriasis, as an immune-mediated inflammatory disease, is often accompanied by AIBDs, especially BP [174]. The risk of concurrent psoriasis in patients with BP is about 2.5 times that of the general population [175]. In traditional treatment, glucocorticoids used for BP may aggravate psoriasis after withdrawal, while some anti-psoriasis treatments, such as biological agents and phototherapy, may induce or aggravate BP, thus increasing the complexity of comorbidity management [176]. There are currently no standard treatment guidelines for these comorbidity cases. The multi-indication drugs described below are primarily used in psoriasis, but due to the shared immune mechanisms, such as Th17 and IL-23 pathways, they may be extended to the application of psoriasis-AIBD comorbidity (Figure 3).

### 7.1. TNF-α Inhibitor

TNF-α is a central mediator of inflammation in psoriasis. A range of TNF-α inhibitors (including adalimumab, etanercept, infliximab, certolizumab pegol, and golimumab) have been established as effective and safe treatment options for moderate-to-severe psoriasis and psoriatic arthritis [40,177,178]. Multiple case reports have suggested that infliximab and etanercept may hold potential as therapeutic options for patients with PV [179,180,181,182]. Sulfasalazine and pentoxifylline can also reduce the serum TNF-α level and treat PV effectively [183].

When TNF-α inhibitors are used in patients with concomitant psoriasis and AIBDs, a paradoxical effect has been observed: agents such as etanercept, adalimumab, and infliximab, while capable of improving disease manifestations in some cases, may also trigger the onset of BP, PV, or psoriasis, leading to highly variable individual responses [184,185,186,187,188,189]. The mechanism of inducing BP and PV may involve the destruction of immune tolerance by TNF-α inhibitors, resulting in B cell activation, Treg dysfunction, and autoantibody production [189]. Therefore, during the clinical use of such drugs, it is necessary to closely monitor the patients’ autoantibody levels and skin lesion changes for the early identification and management of paradoxical immune reactions.

### 7.2. Secukinumab

Secukinumab, a fully human monoclonal antibody targeting IL-17A, is primarily used in patients with moderate-to-severe psoriasis [177] and has shown sustained efficacy [190,191,192]. However, its use may trigger the onset of AIBDs in some patients, including BP [193,194,195,196] and pemphigus erythematosus [197]. This may be because the targeted intervention of secukinumab disrupts the balance of shared immune pathways between psoriasis and AIBDs, leading to the emergence of new autoimmune responses [197]. Although the incidence of such events is relatively low, their occurrence highlights the importance of clinical monitoring.

In the cases of BP successfully treated with secukinumab [198,199,200], the drug was found to reduce circulating anti-BP180 antibody levels and achieve sustained complete remission [198,199]. Among patients with concomitant psoriasis and BP receiving secukinumab, some achieved complete remission of both conditions, while others experienced worsening of BP [201]. This suggests that the role of the IL-17 pathway in BP is complex. Individual risks should be assessed and disease progression closely monitored during clinical application.

### 7.3. Ixekizumab

Ixekizumab is a humanized IgG4 monoclonal antibody that selectively binds to and neutralizes IL-17A. It is indicated for the treatment of moderate-to-severe plaque psoriasis. In recent years, ixekizumab has been shown to be effective in patients with psoriasis and concomitant BP. Two case reports support ixekizumab’s ability in managing both conditions. A 37-year-old female patient with a 20-year history of psoriasis (previously treated with corticosteroids only) received ixekizumab (160 mg at week 0, then 80 mg at week 2, 4, 6, 8, 10, 12, and thereafter 80 mg every 4 weeks) after developing BP. She subsequently experienced improvement in both diseases within 2 weeks, clinical remission at 12 weeks and sustained remission at 1-year follow-up without adverse events [202]. This case provides a measure of supportive clinical evidence, yet longer follow-up is needed to confirm long-term efficacy and safety. A 63-year-old male patient with an 18-year history of irregularly treated psoriasis developed BP and was treated with intravenous methylprednisolone plus methotrexate. However, his psoriasis exacerbated and new bullae emerged during corticosteroids tapering. He then initiated standard ixekizumab therapy. At week 12, new blisters emerged as the dosing interval was extended. By week 20, he achieved near-complete psoriasis clearance and no new blisters, allowing methotrexate withdrawal and prednisone tapering. This case suggests maintaining corticosteroid dosage when prolonging ixekizumab intervals to minimize disease fluctuation [203].

Conversely, one case reported paradoxical exacerbation of BP following ixekizumab. A 71-year-old male patient with a 60-year history of psoriasis was diagnosed with BP 2 years earlier and was previously treated with prednisolone. After receiving ixekizumab, his psoriasis lesions markedly improved. However, BP flared severely after the second injection. Ixekizumab was immediately stopped, and methylprednisolone plus minocycline was given, with significant improvement at 1 month [196]. This case alerts clinicians to the risk of BP relapse when using IL-17A inhibitors in psoriasis.

### 7.4. Ustekinumab

Ustekinumab is a monoclonal antibody that targets the shared p40 subunit of IL-12 and IL-23, thereby suppressing both Th1 and Th17-mediated inflammatory pathways. It has been approved for the treatment of psoriatic arthritis and moderate-to-severe psoriasis [204,205].

The potential therapeutic role of ustekinumab in BP was investigated through in vitro experiments and a clinical study (NCT04117932). The results indicated that ustekinumab could induce long-term remission in some patients with BP, although it may be associated with serious adverse events [204]. A case of using ustekinumab to treat a patient with comorbid psoriasis and relapsing BP suggested that ustekinumab represented a potential option warranting further investigation for simultaneously controlling both conditions and achieving seroconversion of BP-specific antibodies to negative [206]. Successful use of ustekinumab has also been documented in cases of psoriasis comorbid with other AIBDs [207].

Several cases have reported that patients with psoriasis developed BP after treatment with ustekinumab [194,208], and some of them tested negative for specific autoantibodies [209], or experienced recurrent BP episodes [210]. Possible explanations for this phenomenon include: ustekinumab’s blockade of the Th1/Th17 pathway may lead to an immune balance shift toward a Th2 phenotype, which is associated with BP; biologic agents may increase the risk of infection, thereby triggering BP; many of the reported cases had a history of prior anti-TNF-α therapy, suggesting that previous immunomodulation might increase the subsequent risk of developing BP [194,208]. Therefore, the benefits and risks need to be weighed in clinical application, and new or recurrent blisters should be closely monitored.

### 7.5. Guselkumab

Guselkumab, a monoclonal antibody targeting the p19 subunit of IL-23, has demonstrated high and durable efficacy in the treatment of psoriasis, particularly in biologic-naïve patients [211]. While there is abundant evidence supporting the efficacy and safety of guselkumab in psoriasis, data on its use in patients with comorbid AIBDs remain limited. A case of a multirefractory BP patient coexisting with psoriasis and psoriatic arthritis who was successfully treated with guselkumab has been reported [175]. In a similar vein, there was a case in which guselkumab controlled both psoriasis and concurrent anti-p200 pemphigoid [212]. In these two cases, patients achieved complete remission of both psoriatic and bullous lesions within two months. However, guselkumab also carries the risk of inducing BP in patients with psoriasis [213,214]. When drug-induced BP arises, the agent should be promptly discontinued, and appropriate treatment should be initiated to prevent disease progression.

**Table 4:** Landscape of multiple-indication drugs for PV and BP.

Drug	Target	Regulatory /Clinical Status	Study (Phase)	Status	Characteristics	Main Results	Actual (Estimated) Participants	Level of Evidence *
TNF-α inhibitor	TNF-α	FDA-approved for plaque psoriasis, etc.; investigational/off-label for PV	NCT00283712 (Phase 2) [215]	Completed in 2011	Randomized, double-blind, placebo-controlled trial comparing infliximab + prednisone vs. placebo + prednisone in PV	Infliximab and placebo groups showed identical response rates (10% each) at 18 weeks.	20	2
			NCT00135720 (Phase 2)	Completed in 2007	Randomized, double-blind, placebo-controlled study evaluating etanercept in PV	No results posted.	(12)	-
Secukinumab	IL-17A	FDA-approved for plaque psoriasis, etc.; investigational/off-label for BP	Case report evidence only [169,193,195,196,198,199,200,201,216,217]					5
Ixekizumab	IL-17A	FDA-approved for plaque psoriasis, etc.; investigational/off-label for BP	Case report evidence only [196,202,203,218,219]					5
Ustekinumab	The shared p40 subunit of IL-12 and IL-23	FDA-approved for plaque psoriasis, etc.; investigational/off-label for BP	NCT04117932 (Phase 2) [204]	Completed in 2023	Open-label, single-arm, multicenter, proof-of-concept study evaluating ustekinumab + an 8-week superpotent topical corticosteroids regimen in BP	40% achieved sustained corticosteroids-free CR at 28 weeks; 5 relapsed after 16 weeks; 7 SAEs reported.	15	4
Guselkumab	The p19 subunit of IL-23	FDA-approved for plaque psoriasis, etc.; investigational/off-label for BP	Case report evidence only [175,213,214]					5

* The levels of evidence are determined according to the Oxford Centre for Evidence-Based Medicine grading criteria (2011). TNF-α—tumor necrosis factor-α; IL—interleukin.

## 8. Challenges and Conclusions

This review summarizes the key advances in research on targeted therapies for PV and BP. By precisely modulating core pathogenic mechanisms, such as autoantibody production and cytokine-driven inflammation, these novel agents are reshaping the therapeutic landscape. At present, targeted therapies have begun to address the major limitations of conventional treatment, offering options that combine high efficacy with remarkable specificity.

Biological agents play an important role in the treatment of immune-related dermatological conditions. However, they also possess drawbacks that cannot be overlooked, among which two aspects deserve special attention. The first is “paradoxical phenomenon”, a situation in which agents used to treat skin diseases unexpectedly trigger or worsen immune-mediated disorders. This phenomenon primarily involves TNF-α, IL-12/IL-23, and IL-17 inhibitors, with TNF-α inhibitors accounting for the majority of reported cases (approximately 91.2%) [220]. Psoriasis is the most common disease linked to these paradoxical reactions [221]. AIBDs, though relatively rare, have also been documented in case reports, with BP being the most frequent type and often arising in patients with underlying psoriasis [220]. The possible mechanisms are thought to be related to Th1/Th2 imbalance, complex inflammatory networks, and interindividual differences in the immune microenvironment [185,194,220,222]. Clinicians should therefore remain vigilant when using biological agents in patients with psoriasis or those with concomitant psoriasis and AIBDs, and be prepared to discontinue or adjust therapy promptly if a paradoxical phenomenon occurs. The second concern is the phenomenon of “immune drift”, in which biological agents specifically targeting psoriasis or atopic dermatitis pathways inadvertently tip the delicate balance between these two immune response patterns. As a result, agents used to treat psoriasis (such as secukinumab) may trigger eczematous lesions, while those indicated for atopic dermatitis (like dupilumab) can conversely induce psoriasis. This shift in disease phenotype is estimated to occur in approximately 1–10% of patients treated with these agents. The underlying pathogenesis may involve shared genetic backgrounds and inflammatory pathways. Psoriasis is primarily driven by Th1/Th17 responses, whereas atopic dermatitis is characterized by Th2-dominant immunity. When one pathway is suppressed by a biological agent, compensatory upregulation of the cytokine levels in the alternative pathway may occur, leading to a shift in disease expression toward the opposite phenotype. In the context of “immune drift”, treatment selection should therefore prioritize agents with efficacy against both conditions, such as JAK inhibitors [186].

An important and increasingly active dimension in the evolving therapeutic landscape for AIBDs is the repurposing of small-molecule inhibitors and other targeted agents originally developed for oncology or other immune-mediated conditions. Many of the inhibitors reviewed herein, like JAK inhibitors (e.g., tofacitinib, approved for rheumatoid arthritis), BTK inhibitors (e.g., rilzabrutinib, initially designed for immune thrombocytopenia), and PDE4 inhibitors (e.g., apremilast, originally approved for psoriasis), were not primarily conceived for bullous diseases, yet they have shown preliminary efficacy in PV/BP through their modulation of overlapping inflammatory and autoimmune signaling cascades. Similarly, FcRn-targeting agents such as efgartigimod, repurposed from myasthenia gravis, illustrate the translational potential of agents that interfere with IgG recycling. This repurposing strategy confers distinct pragmatic advantages: extensive pharmacokinetic, pharmacodynamic, and safety datasets from previous large-scale trials are readily available, manufacturing processes are already established, and clinical development timelines can be substantially compressed compared with de novo drug discovery. Moreover, the pleiotropic actions of certain kinase inhibitors (like concurrent suppression of B cell receptor signalling, mast cell activation, and downstream cytokine production) may offer synergistic therapeutic benefits that are especially valuable in treatment-refractory patients or those with overlapping immune-mediated comorbidities such as psoriasis. Nevertheless, several obstacles merit careful consideration. Doses and schedules validated for original indications cannot be directly extrapolated to AIBDs, as target engagement, pathological tissue distribution, and immune cell turnover differ substantially among various diseases. Furthermore, safety profiles established in original populations do not adequately reflect the elderly, multimorbid AIBD population, in whom risks of infection, thrombosis, and cardiovascular events are of particular concern. Consequently, successful repurposing in PV and BP requires dedicated dose-optimization studies, patient stratification using predictive biomarkers, and disease-specific pharmacovigilance, rather than reliance on extrapolation from unrelated disease contexts.

With respect to CAAR-T, there are still unresolved issues. Whether lymphodepletion (such as cyclophosphamide, fludarabine) is required for CAAR-T therapy in PV remains an open question. In conventional CAR-T for haematological malignancies, lymphodepleting conditioning creates an immunological niche that promotes infused T cell expansion and persistence, while also directly eliminating some pathogenic B cells. However, this approach carries substantial risks, including cytopenias and neurotoxicity. In the preclinical study of Dsg3 CAAR-T, lymphodepletion was not employed [172,173]. This may be because, unlike B cell malignancies, autoreactive anti-Dsg3 B cells in PV patients only account for a small portion of the total number of B cells; preconditioning may increase the risk of infection, while the original design intention of CAAR-T is to avoid systemic immunosuppression and accurately eliminate pathogenic B cells; moreover, the NOD-scid-γ mice used themselves are severely deficient in T cells, B cells, and natural killer cells. Importantly, the result suggests that infused CAAR-T cells can expand, persist, and specifically eliminate targeted B cells without preconditioning. According to the currently disclosed data of the ongoing phase 1/2 trial (NCT04422912), the subjects also received cell infusion without preconditioning. Whether conditioning offers a net benefit requires further data. Beyond chemotherapeutic depletion, alternative strategies, such as FcRn inhibitors, intravenous immunoglobulin, or plasmapheresis, could also be considered to reduce autoantibody burden and optimize the microenvironment for CAAR-T cells.

Additional limitations of targeted therapies include: the existing targeting pathways are relatively narrow, such as targeting only B cells or inflammatory process; substantial heterogeneity in clinical response, posing challenges for elderly patients and those with comorbidities; and high costs that restrict accessibility. These shortcomings largely stem from an insufficient depth of mechanistic research, lagging translational progress, and a lack of long-term data from large-scale randomized controlled trials, resulting in inadequate evidence to clearly establish efficacy. Furthermore, in studies concerning patients with comorbid psoriasis and AIBDs, it remains to be elucidated whether the administered agents exert a direct effect on the AIBDs themselves or improve the overall disease status indirectly through the control of psoriasis. The specific underlying mechanisms warrant further exploration.

Overall, the challenges focus on reliable efficacy, long-term safety, and the need for personalized application. Potential strategies for improvement are as follows: advancing combination strategies, such as integrating biologics with small-molecule drugs, to simultaneously target antibody production and inflammatory pathways; enhancing personalized treatment and optimizing dosing to minimize adverse effects; exploring innovative formulations, such as topical administration to reduce systemic toxicity; and investigating novel targets, including alternative complement pathways or approaches to restore adhesion protein function.

## Data Availability

No new data were created or analyzed in this study. Data sharing is not applicable to this article.
